# Workshop on Germ Cells

**DOI:** 10.3389/fcell.2018.00157

**Published:** 2018-11-21

**Authors:** Roland Dosch

**Affiliations:** ^1^Institute for Developmental Biochemistry, University Medical Center, Göttingen, Germany; ^2^Institute of Human Genetics, University Medical Center, Göttingen, Germany

**Keywords:** zebrafish (Danio rerio), germ cells, fertility, oogenesis and embryonic development, gene editing, germ cell migration, European zebrafish PI Meeting, Trento Italy

## Abstract

Germ cell research in vertebrates has traditionally been challenging, but recent breakthroughs have overcome technical difficulties, demonstrating and expanding the power of the zebrafish experimental system for their analysis *in vivo*. Exploiting the transparency of the zebrafish embryo, germ cell migration was the first topic that moved the germ cells of this organism into the spotlight of modern research. In recent years, research on teleost germ cells has expanded into additional fields, manifested by a session dedicated to this cell type at the European Zebrafish PI meeting in Trento.

The first talk by **Yaniv Elkouby** (The Hebrew University of Jerusalem, Israel) started with the earliest stages of oogenesis. Addressing these early stages in the adult ovary is challenging and Yaniv established the juvenile zebrafish ovary as an excellent model for early oogenesis (Elkouby, 2017). Using this system, Yaniv pioneered live imaging of follicle formation in zebrafish (Elkouby and Mullins, 2017). In his talk, he showed spectacular movies of chromosomes forming the meiotic *bouquet* arrangement *in vivo*, in which all chromosome telomeres cluster in one location of the inner nuclear membrane. His data showed that the polarity of the *bouquet* predicts the future animal-vegetal axis of the oocyte. He thereby discovered the first cellular markers for oocyte asymmetry during oogenesis (Elkouby et al., 2016), and highlighted the function of the oocyte centrosome in integrating meiosis, oocyte patterning and Balbiani body assembly in the early differentiating oocyte (Elkouby, 2017). Traditionally, zebrafish oogenesis was subdivided into five stages, but Yaniv now identified additional features that characterize the progression of oogenesis. Based on these features, he purified stage-specific oocytes and performed RNA-sequencing, in collaboration with Mary Mullins and Antonio Giraldez. This work identified a differential gene expression signature with dynamic trends in gene ontogeny between stages, providing further molecular insight into early oogenesis.

A recurrent obstacle for studying the role of key regulators during germline development is their genetic depletion. A large proportion of these genes also play crucial roles in the somatic cells or cause early loss of the germline. **Florence Marlow** (Icahn School of Medicine at Mount Sinai in New York, USA) tackled this challenge by establishing a system for germline specific gene editing *via* CRISPR/Cas9. Using specific promoters, she can either activate Cas9 in the entire germline or specifically in the female. As a demonstration of the technology, she recapitulated the *kif5Ba* mutant maternal-effect phenotype previously described by her laboratory (Campbell et al., 2015), but without somatic defects. As this innovative technology allows eliminating somatic side-effects, this interesting approach will have a great impact on future research in the zebrafish germline field.

The process of fertilization has fascinated researchers since its discovery, but the identification of the molecular players in vertebrates has been difficult (Evans, 2012; Miyata et al., 2016). Two talks identifying novel gene functions essential for female fertility open new lines of research in reproductive biology. While a postdoc in Alex Schier's lab at Harvard University, **Andrea Pauli** previously identified hundreds of novel transcripts that were predicted to encode for short proteins (Pauli et al., 2014). In her talk, Andi presented unpublished results from her own lab (Research Institute of Molecular Pathology (IMP) Vienna BioCenter, Austria) concerning one of those candidate proteins that they named Bouncer (Herberg et al., 2018). Bouncer is a conserved GPI-anchored vertebrate protein that localizes to the egg membrane in fish. *Bouncer* mutant female zebrafish are sterile due to a defect in sperm entry. Remarkably, replacing zebrafish Bouncer with Bouncer of Medaka, an evolutionarily distant fish, allowed Medaka sperm to fertilize zebrafish eggs. Thus, Bouncer acts as gate-keeper of the egg during fertilization by allowing conspecific sperm to enter while keeping heterospecific sperm out. These experiments identify Bouncer as a novel, maternal protein, which mediates species-specific fertilization.

In addition to species-specific recognition mechanisms between sperm and egg, in several species including teleosts, fertilization requires a peculiar opening at the surface of the vitelline membrane that facilitates sperm entry and is called the micropyle. The micropyle mechanically prevents polyspermy by restricting the entry of only a single sperm. It is formed by a single specialized follicle cell, the micropylar cell, that grows much bigger than its neighbors and acquires a typical mushroom-shape during oogenesis. To date, hardly anything is known about the molecular pathways controlling micropyle formation. The lab of **Virginie Lecaudey** (Goethe University of Frankfurt, Germany) discovered that females mutant for the Hippo pathway effector Taz/*wwtr1* are sterile, because their oocytes do not form a functional micropyle (Dingare et al., 2018). Indeed, she showed that a Taz antibody specifically labeled the micropylar cell. Taz therefore provides the first marker to molecularly distinguish the micropylar cell from the other follicle cells that surround the oocyte. Using this marker, they could follow the cytoskeletal changes occurring in the prospective micropylar cell itself and at the oocyte animal pole cortex, with which it remains associated. Through identifying this role for the *taz* gene Virginie‘s lab has established a molecular foothold to unravel the cellular changes associated with micropyle formation and provided novel insights into the molecular cause of sterility in zebrafish.

Zebrafish germ cells are specified during embryogenesis by a germline-specific RNP-granule termed germ plasm. The lab of **Roland Dosch** (Georg-August University of Goettingen, Germany) previously identified the Bucky ball (Buc) protein as the first vertebrate germ plasm organizer (Dosch et al., 2004; Marlow and Mullins, 2008; Bontems et al., 2009). Importantly, the amino acid sequence of Bucky ball does not provide any information about its biochemical mechanism, but Roland showed that the germ plasm organizer Oskar from *Drosophila* can, like Buc, promote formation of germ cells in zebrafish (Krishnakumar et al., 2018). Thus, although the *Drosophila* and zebrafish proteins show no protein sequence similarities, zebrafish Bucky ball and *Drosophila* Oskar appear to have similar activities in the embryo, which presumably involve interactions with the same germ plasm proteins and RNAs to promote germline fate. These results identify the first protein pair, which has the same function in different organisms in the absence of any sequence homology. These data thus indicate that germline specification might be more conserved than previously anticipated.

Although primordial germ cells differentiate into only one type of cells, the gametes, the germline is considered to be totipotent as it can give rise to the complete organism in the next generation. This totipotent property should be suppressed in primordial germ cells that are exposed to a range of differentiation signals during their migration. Relevant for the control over germ cell fate, the lab of **Erez Raz** (Muenster University, Germany) had previously identified the Dead end protein as a key regulator of zebrafish germline development. It was later discovered that Dead end also plays a crucial role in the mammalian germline, where mutations in Dead end cause depletion of germ cells and teratomas. In his talk, Erez reported remarkable results that upon knockdown of Dead end, zebrafish germ cells cannot maintain their germline fate (Gross-Thebing et al., 2017). While a small proportion of germ cells undergo apoptosis, most of the *dead end* depleted cells differentiate into somatic cell types of the three germ layers. Indeed, Erez showed data, in which his lab reprogrammed Dead end depleted primordial germ cells into endodermal derivatives by overexpressing Taram-a, which is a constitutively-active TGF-β receptor known to induce endoderm in early embryos (Renucci et al., 1996; Peyriéras et al., 1998). In addition to the contribution toward understanding the process of germ cell fate maintenance, these data highlight the possible use of this intriguing cell population in regenerative therapies.

These interesting presentations spurred lively discussions. The session emphasized the recent contribution of the zebrafish to major technical and conceptual advances in germ cell biology, long sought after since postulated by Weismann in 1895. The spectrum of the talks at the workshop demonstrated that the research field is increasingly expanding but made also clear that many aspects of the zebrafish germline such as spermatogenesis should be added to future meetings. This trend was also visible in other sessions at this European Zebrafish PI meeting, where additional studies of germline biology were presented such as the talk of René Ketting (IMB, Mainz, Germany) on the activation of the piRNA pathway in primordial germ cells. Overall, the germ cell workshop demonstrated the potential of the zebrafish model in expanding our knowledge of vertebrate germ cell specification, fate maintenance, and gamete differentiation and function. The positive momentum present at the meeting promises further exciting discoveries in the future, through and beyond the next European Zebrafish PI Meeting in 2021.

## Author contributions

The author confirms being the sole contributor of this work and has approved it for publication.

### Conflict of interest statement

The author declares that the research was conducted in the absence of any commercial or financial relationships that could be construed as a potential conflict of interest.
